# Antigen-Specific Gene Therapy after Immunisation Reduces the Severity of Collagen-Induced Arthritis

**DOI:** 10.1155/2013/345092

**Published:** 2013-11-26

**Authors:** Tove Eneljung, Sara Tengvall, Pernilla Jirholt, Louise Henningsson, Rikard Holmdahl, Kenth Gustafsson, Inger Gjertsson

**Affiliations:** ^1^Department of Rheumatology and Inflammatory Research, University of Gothenburg, P.O. Box 480, 405 30 Gothenburg, Sweden; ^2^Medical Inflammation Research, Karolinska Institute, 171 77 Stockholm, Sweden; ^3^Molecular Immunology Unit, Institute of Child Health, UCL, 30 Guilford Street, London WC1N 1EH, UK

## Abstract

Reestablishment of tolerance induction in rheumatoid arthritis (RA) would be an optimal treatment with few, if any, side effects. However, to develop such a treatment further insights in the immunological mechanisms governing tolerance are needed. We have developed a model of antigen-specific tolerance in collagen type II (CII) induced arthritis (CIA) using lentivirus-based gene therapy. The immunodominant epitope of CII was inserted into a lentivirus vector to achieve expression on the MHC class II molecule and the lentiviral particles were subsequently intravenously injected at different time points during CIA. Injection of lentiviral particles in early phases of CIA, that is, at day 7 or day 26 after CII immunisation, partially prevented development of arthritis, decreased the serum levels of CII-specific IgG antibodies, and enhanced the suppressive function of CII-specific T regulatory cells. When lentiviral particles were injected during manifest arthritis, that is, at day 31 after CII immunisation, the severity of arthritis progression was ameliorated, the levels of CII-specific IgG antibodies decreased and the proportion of T regulatory cells increased. Thus, antigen-specific gene therapy is effective when administered throughout the inflammatory course of arthritis and offers a good model for investigation of the basic mechanisms during tolerance in CIA.

## 1. Introduction

A hallmark of autoimmune diseases such as rheumatoid arthritis (RA) is immune responses directed against self-antigens and hence loss of tolerance against self. Today's treatment for RA is based on a combination of general immunosuppression and highly efficient specific biologicals, for instance, TNF (tumor necrosis factor) inhibitors [1]. However, about one-third of patients with active RA do not respond to available treatments or suffer from severe side effects [2, 3]. An alternative strategy of ameliorating inflammation in autoimmune diseases could be to reestablish tolerance. An optimal tolerance induction would abolish the autoimmune inflammation but still retain a capacity of the immune system to respond to pathogens.

Collagen type II (CII) is recognized as an autoantigen in RA, and CII-induced arthritis (CIA) in mice is a widely used animal model of RA. Autoreactive T cells directed against the CII amino acid (aa) sequence 259–270 are present in both RA and CIA [4–10], as are antibodies recognising CII, and in RA patients their presence predicts a more destructive disease [11]. In animal models of autoimmune diseases, the autoantigen is used to induce disease but can also be used as a tolerance-inducing antigen (tolerogen); for example, administration with soluble CII peptides or whole protein can suppress the development of CIA [12–14]. However, the use of soluble tolerogenic peptides has disadvantages. First, repeated injections of the peptides can cause severe side effects such as anaphylactic reactions or disease flares [15–17]. Second, the effect is limited due to rapid degradation of the peptide, and thus repeated or continuous administration of high doses of the tolerogen is necessary [13, 15, 18–23]. In order to minimize these limitations, modified CII peptides have been used in complex with major histocompatibility complex II (MHC II) molecules, fused with choleratoxin or administered as a DNA vaccine with improved results [24–26].

Another approach to induce tolerance in mouse models of RA is by gene therapy. The lentiviral-based gene therapy system is advantageous since it has low immunogenicity *per se* and efficiently integrates the gene of interest into the host genome [27, 28]. The peptide expressed as a result of gene integration is presented on MHC II without simultaneous activation of antigen presenting cell (APC), a feature well suited for tolerance induction [29]. In addition, gene therapy gives a longstanding effect as the expressed protein has the potential to be continuously produced [30, 31]. Thus, the lentiviral system offers a potentially ideal approach to induce tolerance in order to explore tolerogenic mechanisms in the inflammatory phases of CIA.

In a previous study we show that prophylactic gene therapy using lentiviral particles encoding the invariant chain fused to the immunodominant CII peptide (LNT-Ii-CII) induces antigen-specific tolerance and suppresses the development of arthritis [30]. However, it is not known whether injection of these lentiviral particles is efficient in the inflammatory phases of CIA, which was thus the aim of the present study.

## 2. Methods

### 2.1. Generation of Constructs and Production of Lentiviral Particles

**Figure 1:**
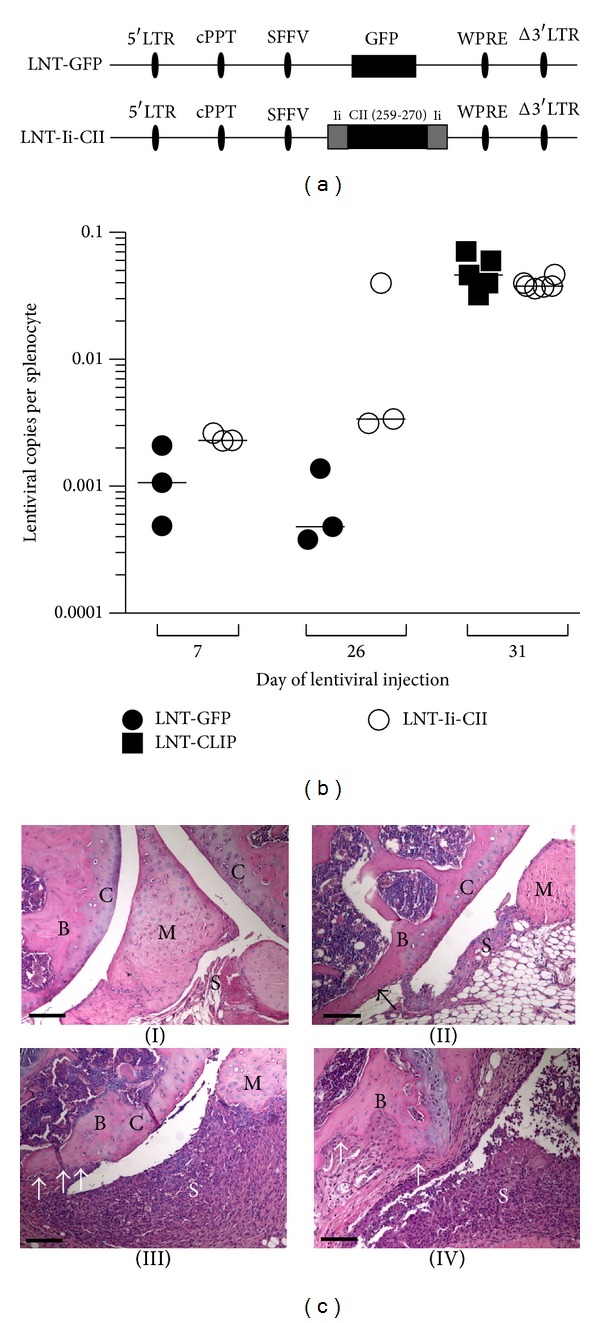
Lentiviral gene constructs, design of experiments, integration, and histopathological scoring. (a) Lentiviral constructs: LNT-GFP and LNT-Ii-CII. LTR: long terminal repeat; cPPT: central polypurine tract; SFFV: spleen focus-forming virus promoter; GFP: green fluorescent peptide; WPRE: woodchuck posttranscriptional regulatory element; Ii: invariant chain; CII: collagen type II peptide amino acids 259–270. (b) Integration of lentiviral vectors analysed in splenocytes at termination of experiments after lentiviral treatment at day 7, 26, or 31 after immunisation. The number of lentiviral particles is expressed as integrated lentiviral particle per cell. Data were analysed by Mann-Whitney *U* test. Closed circles represent LNT-GFP, squares represents LNT-Ii-CLIP, and open circles represent LNT-Ii-CII treated mice. (c) Histopathological scoring in the knee joints at day 56 after CII immunization. Mice were injected with LNT-GFP at day 26. (I) Healthy joint; (II) synovitis and bone erosions grade 1; (III) synovitis grade 3 and bone and cartilage destruction grade 2; (IV) synovitis and bone and cartilage destruction grade 3. S = synovia, C = cartilage, B = bone, M = meniscus, and bone and cartilage destruction are marked with arrows. Scale bar, 100 *μ*m.

A detailed description of the generation of control construct, pHR'SIN-cPPT-SEW (LNT-GFP), control construct LNT-Ii-CLIP, and treatment construct LNT-Ii-CII driven by the spleen focus-forming virus promoter has been described previously [30, 32]. In summary, the lentiviral construct LNT-Ii-CII (Figure 1(a)) contains the rat sequence coding the immunodominant T cell CII epitope, amino acids (aa) 259–270, fused into the class-II associated invariant chain-associated peptide (CLIP) position of MHC II stabilising protein invariant chain (Ii) which ensures presentation of CII peptide on MHC II molecules. As control vectors we have used the green fluorescent peptide (LNT-GFP) (Figure 1(a)) or the construct containing the gene coding the naturally occurring CLIP peptide Ii (LNT-Ii-CLIP) (Figure 4(a)).

Vesicular stomatitis virus G (VSV-G) pseudotyped lentivirus was produced by transient transfection of 293 FT cells with the self-inactivating transfer vector plasmid (LNT-GFP, LNT-Ii-CLIP, or LNT-Ii-CII), the multideleted packaging plasmid pCMVΔR8.74, and the VSV-G envelope pMD.G2 by calcium phosphate coprecipitation (ProFection Mammalian Transfection System, Promega Biotech AB, Stockholm, Sweden) and concentrated by ultracentrifugation at 90,000 g. The pellets were resuspended in PBS (phosphate-buffered saline) containing 2% FCS (fetal calf serum, Sigma-Aldrich, St. Louis, MO, USA) and stored at −80°C.

### 2.2. Lentiviral Particle Titration

Viral titers were determined on the HeLa cells (American Type Culture Collection, Manassas, VA, USA). The expression of GFP was analysed directly using flow cytometry and the expression of LNT-Ii-CLIP and LNT-Ii-CII were determined after intracellular staining of Ii (FITC rat anti-mouse CD74, clone In-1, BD Biosciences, San Jose, CA, USA) using a FoxP3/Transcription factor staining buffer set (eBioscience, Inc., San Diego, CA, USA) 3 days after transduction. The frequency of positive cells was analysed using flow cytometry on a BD FACSCalibur instrument (BD, New Jersey) and analysed by FlowJo Software (Tree Star Inc., Ashland, OR, USA).

### 2.3. Induction of Arthritis and Intravenous Injection of Lentivirus

**Figure 2:**
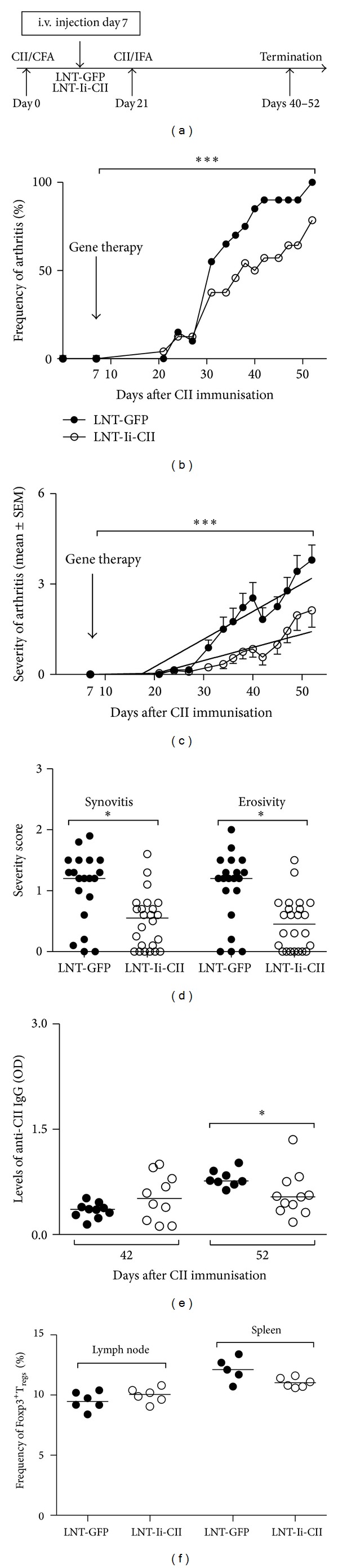
Clinical and histopathological development of arthritis, serum levels of anti-CII IgG, and T regulatory cells after i.v. injection of lentiviral particles at day 7 after CII immunisation. (a) Design of experiments. Immunisation at day 0 with collagen type II (CII) in complete Freund's adjuvants (CFA), lentiviral injection with LNT-GFP or LNT-Ii-CII at day 7 after CII immunisation, booster at day 21 with CII in incomplete Freund's adjuvants (IFA), and termination of experiments at days 42–63. (b) The clinical frequency of arthritis. (c) The clinical severity of arthritis (LNT-GFP days 0–40 *n* = 24, days 42–52 *n* = 14, LNT-Ii-CII days 0–40 *n* = 20, and days 42–52 *n* = 10). (d) The severity of histopathological synovitis and bone-cartilage erosivity. (e) Serum levels of CII-specific IgG antibodies at days 42 and 52. (f) The frequency of CD4^+^CD25^+^Foxp3^+^  T_regs_ in spleen and lymph node at termination of experiment. Closed circles represent LNT-GFP and open circles represent LNT-Ii-CII treated mice. In Figure 2(b) the *P* value is calculated using logistic regression, in Figure 2(c) the *P* value is calculated using linear regression, and in Figures 2(d), 2(e), and 2(f) the *P* value is calculated using Mann Whitney *U* test. *P < 0.05 and **P < 0.01.

**Figure 3:**
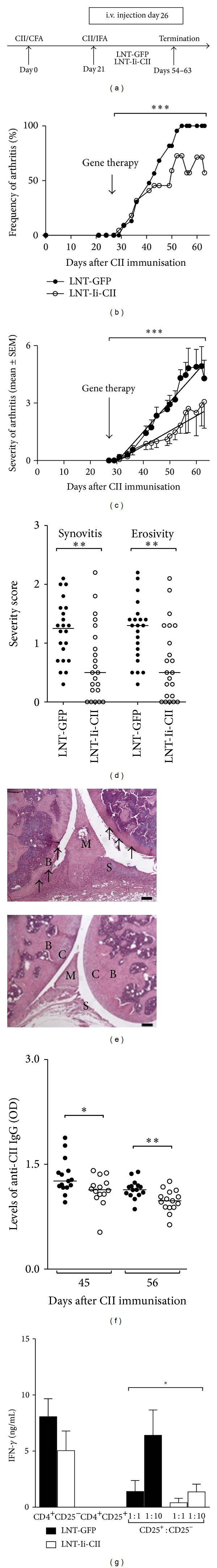
Clinical and histopathological development of arthritis, serum levels of anti-CII IgG, and T cell response after i.v. injection of lentiviral particles at day 26 after CII immunisation. (a) Design of experiments. Immunisation at day 0, lentiviral injection with LNT-GFP or LNT-Ii-CII at day 26 after CII immunisation, booster at day 21, and termination of experiments at days 54–63. (b) The clinical frequency of arthritis. (c) The clinical severity of arthritis (LNT-GFP days 0–43 *n* = 23, days 45–54 *n* = 22, days 56–63 *n* = 7, LNT-Ii-CII days 0–54 *n* = 22, and days 56–63 *n* = 7). (d) The severity of histopathological synovitis and bone-cartilage erosivity. (e) Histopathological findings in the knee joints at day 56 after CII immunization in a mouse injected with LNT-GFP (top panel) that shows synovitis and bone and cartilage destruction grade 2. The bottom panel shows the knee joint from an LNT-Ii-CII mouse with a nonarthritic knee joint at the same time point. S = synovia, C = cartilage, B = bone, M = meniscus, and bone and cartilage destruction are marked with arrows. Scale bar, 100 *μ*m. (f) Serum levels of CII-specific IgG antibodies at days 45 and 56. (g) Suppressive capacity of T cells measured as levels of IFN-**γ** in supernatants from cocultures of T cell subsets and CD11c^+^ cells. The two first bars represent supernatants from cocultures containing CD4^+^CD25^−^ and CD11c^+^ cells, the following two CD4^+^CD25^+^ cells and CD11c^+^ cells, and finally 4 bars represent a mix of CD4^+^CD25^−^ and CD4^+^CD25^+^ in a ratio of 1 : 1 or 1 : 10 cocultured with CD11c^+^ cells. Closed circles represent LNT-GFP and open circles represent LNT-Ii-CII treated mice. In Figure 3(b) the *P* value is calculated using logistic regression, in Figure 3(c) the *P* value is calculated using linear regression, and in Figures 3(d) and 3(f) the *P* values are calculated using Mann Whitney *U* test. In Figure 3(g) black bars represent cells from LNT-GFP treated mice and white bars represent cells from LNT-Ii-CII treated mice. The *P* value is calculated using two-way ANOVA comparing the increasing IFN-**γ** levels from T cell cocultures from LNT-GFP mice in ratios from 1 : 1 to 1 : 10 with respect to IFN-**γ** levels from cells from LNT-Ii-CII mice in ratios from 1 : 1 to 1 : 10. *P < 0.05 and **P < 0.01.

**Figure 4:**
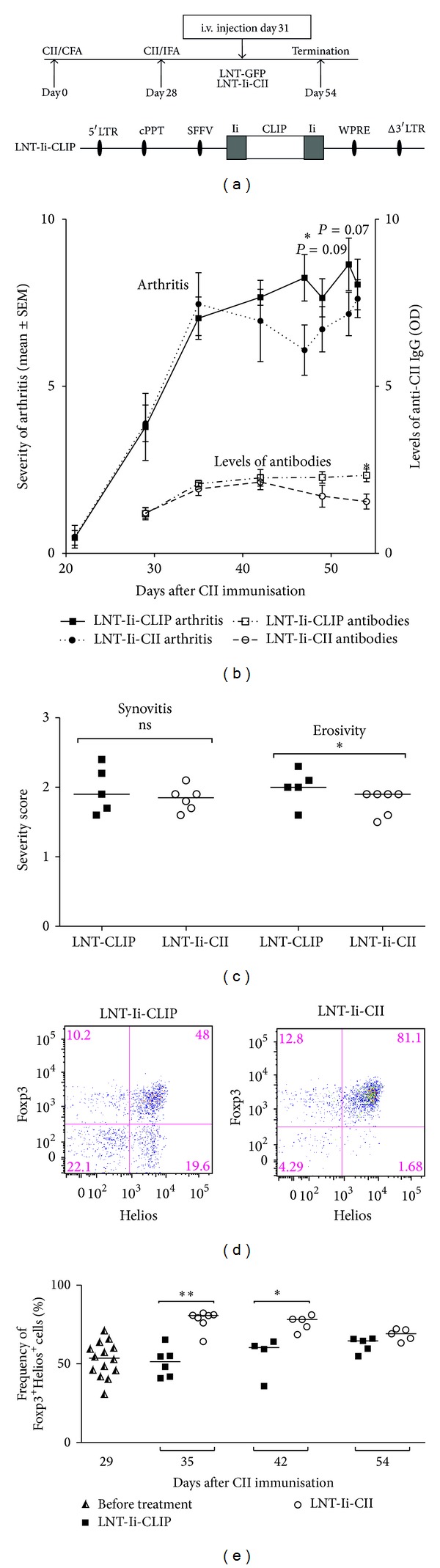
Lentiviral treatment at day 31. (a) Design of experiment: Immunisation at day 0, booster at day 28, lentiviral injection with LNT-Ii-CLIP or LNT-Ii-CII at day 31 after CII immunization, and termination of experiments at day 54. LNT-Ii-CLIP construct, CLIP: class-II associated invariant chain peptide. (b) The clinical severity of arthritis (left *y*-axis) and levels of CII-specific antibodies (right *x*-axis). (c) The severity of histopathological synovitis and bone-cartilage erosivity (LNT-Ii-CLIP *n* = 5, LNT-Ii-CII days *n* = 6). (d) The gating strategy of CD4^+^CD25^+^ and subsequently Foxp3^+^ Helios^+^ cells. (e) The frequency of CD4^+^CD25^+^Foxp3^+^ Helios^+^  T_regs_ in blood at indicated time points after i.v. injection of LNT-Ii-CLIP or LNT-Ii-CII at day 31. The *P* values are calculated using Mann Whitney *U* test. *P < 0.05 and **P < 0.01.

Male DBA/1 mice (Taconic, Ry, Denmark) aged 7–10 weeks were maintained (10 per cage) under standard conditions of temperature and light. At day 0 the mice were immunised subcutaneously with rat CII in complete Freund's adjuvants (CFA) (Sigma-Aldrich) and boosted with rat CII in incomplete Freund's adjuvants (IFA) (Sigma-Aldrich) at day 21 (when lentiviral treatment was administered at day 7 or 26) or boosted at day 28 (when lentiviral treatment was administered at day 31). The change in time for booster, in the experiment in manifest arthritis, was done due to a change in ethical legislation. Lentiviral particles, LNT-Ii-CII or control virus (5 × 10^6^ lentiviral particles/mouse), were injected i.v. in one of the tail veins at one of three time points (Figures 2, 3, and 4(a)): (1) in the early phase of CIA, 7 days after CII immunisation; (2) around onset of arthritis, 26 days after CII immunization; or (3) in manifest arthritis, 31 days after CII immunisation. Blood, spleen, draining lymph nodes, and paws were taken for analysis at indicated time points. Experiments when lentiviral injection was given at day 7 or 26 were repeated and, when possible, results were pooled. Permission from the local animal research ethics committee, in accordance with national animal welfare legislation, was obtained for all the mice experiments. Mice were handled in accordance with the Gothenburg Ethical Committee on Animal Experiments, Gothenburg University, and the ethical committee has approved of this study (ethical permission number 105-2009).

### 2.4. Detection of Transgene Integration after Intravenous Injection of Lentivirus

The lentiviral transgene constructs contain the woodchuck posttranscriptional regulatory element (WPRE) in the integrating part of the lentiviral backbone. DNA was prepared from spleen at termination of experiments at days 52–56, using the QIAamp DNA mini kit (Qiagen, Solna, Sweden) according to the manufacturer's instructions. The WPRE was amplified with forward primer 5′-GGCACTGACAATTCCGTGGT-3′, reverse primer 5′-AGGGACGTAGCAGAAGGACG-3′ (Sigma-Aldrich), and the probe 5′-FAM-ACGTCCTTTCCATGGCTGCTCGC-TAMRA-3′ (Applied Biosystems, CA, USA) [28]. The WPRE copy number was normalised to exon 5 of the mouse *titin* gene, which was amplified with forward primer 5′-AAAACGAGCAGTGACGTGAGC-3′, reverse primer 5′-TTCAGTCATGCTGCTAGCGC-3′ (Sigma-Aldrich), and the probe 5′-FAM-TGCACGGAAGCGTCTCGTCTCAGTC-TAMRA-3′ (Applied Biosystems). The analysis was performed using the 7500 Real Time PCR System (Applied Biosystems). The lentiviral construct could be detected in all analyzed mice at termination of the experiment (Figure 1(b)).

### 2.5. Clinical and Histological Evaluation of Arthritis

Clinical arthritis and severity was assessed by an evaluator blinded to the treatment groups [30]. Finger/toe and ankle/wrist joints were inspected and arthritis was defined as visible erythema and/or swelling. To evaluate the severity of arthritis, a clinical scoring (arthritic index) was carried out using a system where macroscopic inspection yielded a score of 0–3 points for each limb. We define our scoring system as follows: 0: no arthritis, 1: mild arthritis (mild swelling and a subtle erythema of the evaluated joint), 2: moderate arthritis (moderate swelling and a more pronounced erythema compared to score 1), 3: severe arthritis (profound swelling and erythema). The total score per animal and time point is calculated by adding up the scores from all four paws. Histopathologic examination of the joints was performed at termination of experiments as earlier described [30]. Tissue sections from fore and hind paws were cut after routine fixation, decalcification, and paraffin embedding and stained with hematoxylin-eosin. All the slides were coded and evaluated by two blinded observers. The specimens were evaluated with regard to synovial hypertrophy, pannus formation, and cartilage-subchondral bone destruction. The degree of synovitis and destruction in every joint concerning finger/toes, wrists/ankles, elbows, and knees was assigned a score from 0 to 3 (Figure 1(c)). Occasionally, one paw was missing in the histological sections or embedded in such a way that it was impossible to evaluate the degree of synovitis and bone-cartilage destruction. Therefore, the total score per mouse was divided by the number of joints evaluated.

### 2.6. Analysis of CD4^+^CD25^+^ T Regulatory Cell Number in Lymph Nodes and Spleen after Lentiviral Administration at Day 7

Draining lymph nodes and spleen were examined at day 52 after CII immunisation from mice that received lentiviral injection with LNT-GFP (*n* = 6) or LNT-Ii-CII (*n* = 6) at day 7. After single cell preparations were made, cells were counted (Nucleocounter, ChemoMetec AS, Denmark) and quantification of CD4^+^CD25^+^  T_regs_ in cell suspension was performed by flow cytometry using FACSCanto II (BD Biosciences). To avoid nonspecific binding via Fc-receptor interactions, cells were incubated with Fc-block (clone 2.4G2, BD Biosciences) followed by surface staining with Pacific Blue-labeled anti-CD4 (clone RM4-5, BD Biosciences) and APC-labeled anti-CD25 (clone 3C7, BD Biosciences). Cells were permeabilised and fixated before intracellular FITC-labeled anti-FoxP3 (clone FJK-16s, eBioscience) staining using FoxP3/Transcription Factor Staining Buffer set (eBioscience). An intracellular isotype antibody was used as a control for intracellular FoxP3-staining. Analysis was performed by FlowJo Software (Tree Star Inc., Ashland, OR, USA) and gates were set according to fluorochrome minus one (FMO) settings [33].

### 2.7. CII Reactivity in T Cells from Lymph Nodes and Spleen from Mice Administered Lentivirus at Day 26 after CII Immunisation

#### 2.7.1. Purification of Lymph Node Cells and Sorting of T Cells Subsets from Spleen

Single-cell suspensions were made at day 52 from draining lymph nodes and spleen from mice lentivirally injected at day 26 after immunisation. Two to three lymph nodes or 2-3 spleens from each treatment group were pooled to obtain sufficient numbers of cells. The splenocytes were separated into CD4^+^ T cells, CD4^+^CD25^+^  T_regs_, and CD4^+^CD25^−^ T effector cells using a CD4^+^CD25^+^ T cells enrichment kit (Easy Sep, Stemcell, Grenoble, France).

#### 2.7.2. Sorting and CII Stimulation of CD11c-Positive Splenocytes

CD11c-positive cells were purified from a single-cell suspension of splenocytes obtained from naive DBA/1 mice (Easy Sep kit, Stemcell) and plated in 24-well plates in complete Iscove's medium at a concentration of 1 × 10^6^ cells/mL. These CD11c-positive cells were pulsed with 100 *μ*g/mL of denatured CII and incubated overnight in 37°C.

#### 2.7.3. Coculture of Cell Subsets

The T cell subsets from spleen, were cocultured, at a concentration of 4 × 10^5^ cells/well, with CII-pulsed CD11c cells. The T cell subsets were either put in the coculture alone or as a mixture of CD4^+^CD25^−^ cells and CD4^+^CD25^+^ cells in ratios of 1 : 1 or 1 : 10. Supernatants were collected after 96 hours and stored at −20°C for later ELISA analysis of IFN-**γ** levels (R&D Systems, Abingdon, UK).

### 2.8. Levels of Anti-CII and Total IgG Antibodies in Serum

Levels of CII-specific total IgG and the following subclasses: IgG1, IgG2a, and IgG2b were measured at different time points during experiments (Figures 2(e), 3(e), and 4(b)). Briefly, rat CII or goat anti-mouse polyclonal IgG antibodies (Jackson Immunology Research, Suffolk, England) was used as coating and 2% BSA (Sigma-Aldrich) for blocking. Serum samples were serially diluted from 1/7500 to 1/202 500 and the CII-specific IgG was detected by biotinylated rat anti-mouse antibodies (Serotec, Oxford, UK, and AH Diagnostics, Stockholm, Sweden). The total IgG levels in serum, at termination of experiment from mice treated at day 26, was detected by a biotinylated goat anti-mouse IgG (Southern Biotechnology, Alabama, USA). The plates were read in Spectra Max 340 PC (Molecular Devices) at 405 nm or at 450 nm and corrected for 650 nm. Data were expressed as optical density (OD).

### 2.9. Quantification of Thymic CD4^+^CD25^+^ T_**regs**_ in Blood after Lentiviral Administration at Day 31

In the experiment when LNT-GFP, LNT-Ii-CLIP, or LNT-CII was administered at day 31, blood was taken at days 29, 35, 42, and 54 after CII immunisation. Blood was centrifuged at 300 g and red blood cells were lysed using lysis buffer containing EDTA and HCO_3_. Cells were stained for surface markers CD4 (V450-labeled, clone RM4-5, BD Biosciences) and CD25 (FITC-labeled, clone 3C7, BD Biosciences). After permeabilization and fixation the cells were stained for intracellular APC-labeled Helios (clone 22F6, eBioscience) and PE-labeled FoxP3 (clone NRRF-30, eBioscience). Isotype controls were used for intracellular markers. Samples were analysed using FACSCanto II (BD Biosciences) and analysed by FlowJo Software (Tree Star Inc.).

### 2.10. Statistical Analysis

Statistical analysis was performed using GraphPad Prism (La Jolla, CA, USA). Statistical differences between independent groups were calculated using the nonparametric Mann-Whitney *U* test or Fisher's exact probability test. Two-way ANOVA was used when comparing reactivity from cocultures of T cells at different ratios. Logistic regression was used to compare frequency of arthritis between groups and linear regression was used to compare development of severity of arthritis between treatment groups. *P* < 0.05 was considered significant.

## 3. Results

### 3.1. Gene Therapy at Day 7 after CII Immunisation Reduces Progression of CIA and Serum Levels of CII-Specific IgG Antibodies

In order to investigate if gene therapy could be used in the inflammatory phases of CIA we treated mice with an i.v. injection of lentiviral particles at different time points. Integration of the vector could be verified in splenocytes at termination irrespective of when it was administered (Figure 1(b)). First, lentiviral particles were injected in the early phase of CIA—7 days after CII immunisation (Figure 2(a)). At this time point injection of LNT-Ii-CII but not LNT-GFP lentiviral particles reduced both the frequency of arthritis (Figure 2(b)) and the severity of clinical arthritis (Figure 2(c)). Importantly, LNT-Ii-CII treated mice showed a less pronounced synovitis and bone-cartilage destruction compared to LNT-GFP treated controls (Figure 2(d)). Thus, LNT-Ii-CII treatment administered early in CIA efficiently suppresses the development of arthritis regarding both incidence and severity of arthritis, as well as reduced bone-cartilage erosivity.

To further explore the underlying mechanisms behind the difference in development of arthritis between the groups we investigated the serum levels of CII-specific IgG antibodies. Treatment with LNT-Ii-CII at day 7 significantly decreased CII-specific IgG levels at day 42 compared to control mice (Figure 2(e)).

Since T_regs_ are known to play a key role in maintaining tolerance, we investigated the frequency and function of T cells in animals from the different treatment groups. The frequency of CD4^+^CD25^+^Foxp3^+^  T_regs_ in draining lymph nodes and spleen was similar at the end of the experiment whether mice had been treated with LNT-Ii-CII or LNT-GFP at day 7 after CII immunisation (Figure 2(f)).

### 3.2. Gene Therapy Administered at Day 26 after CII Immunisation Reduces the Progression of CIA and the Serum Levels of CII-Specific IgG Antibodies and Increases the Suppressive Function of T Regulatory Cells

Lentiviral particles were also injected i.v. around onset of arthritis—26 days after CII immunisation (Figure 3(a)). Also at this time point, i.v. injection of LNT-Ii-CII but not LNT-GFP lentiviral particles reduced both the frequency (Figure 3(b)) and severity of clinical arthritis (Figure 3(c)). The clinical findings were confirmed by histological evaluation that revealed reduced synovitis and bone-cartilage destruction in the LNT-Ii-CII group compared with controls (Figures 3(d) and 3(e)).

Treatment with LNT-Ii-CII at day 26 also caused significantly decreased serum levels of CII-specific IgG at days 45–56 compared to control mice (Figure 3(f)). To rule out the possibility of a general decrease in IgG antibodies in LNT-Ii-CII treated mice, levels of total IgG antibodies in serum were analysed and showed similar levels in both treatment groups (data not shown).

The frequency of CD4^+^CD25^+^Foxp3^+^  T_regs_ in draining lymph nodes and spleen was similar between the LNT-Ii-CII and control groups (data not shown). Our next step was to investigate if differences in functional properties of T cells contribute to the treatment effect of LNT-Ii-CII in CIA. Splenic T cells from mice injected with lentiviral particles at day 26 after CII immunisation were sorted into CD4^+^CD25^−^ T effector and CD4^+^CD25^+^  T_regs_ and cocultured with CII-pulsed CD11c^+^ cells alone or at 1 : 1 or 1 : 10 ratios (T effectors : T_regs_). The suppressive capacity of T_regs_ on T effector cells was measured as IFN-**γ** production, where decreased levels indicate suppression. When the proportion of T regulatory cells was increased 10 times from 1 : 1 (T effector : T_regs_) to 1 : 10, the levels of IFN-**γ** decreased in cocultures from LNT-Ii-CII mice compared with LNT-GFP controls (Figure 3(g)). Thus, the splenic CD4^+^CD25^+^  T_regs_ from LNT-Ii-CII treated mice have an improved antigen-specific suppressive capacity compared to T_regs_ from LNT-GFP treated controls.

### 3.3. Gene Therapy Administered in Manifest CIA and Its Effects on Arthritis, T Cell Subsets, and Autoantibody Levels

In order to explore the possibility to use gene therapy to induce tolerance in manifest arthritis, we delayed the injection of lentiviral particles until all animals had developed arthritis, that is, at day 31 after immunisation (Figure 4(a)). In this experiment the treatment vector LNT-Ii-CII was compared to a control vector, LNT-Ii-CLIP, coding for the naturally occurring class-II associated invariant chain-associated peptide, CLIP (Figure 4(a)).

LNT-Ii-CII treatment at day 31 did not decrease the frequency of arthritis (data not shown) while the clinical severity was slightly suppressed compared to LNT-Ii-CLIP controls (Figure 4(b)), supported by reduced erosivity in the LNT-Ii-CII group (Figure 4(c)). The reduced clinical severity of arthritis coincided with a gradual decrease in serum levels of IgG CII antibodies determined at days 29, 35, 42, 49, and 54 (Figure 4(b)).

Because the function of T_regs_ was affected after lentiviral treatment at day 26, we wanted to investigate the characteristics and the origin of T_reg_ cell subsets in mice treated in manifest phase (day 31) of CIA. To be able to discriminate between natural thymic and peripherally induced T_regs_, we stained peripheral blood T cells for expression of Helios, known to be upregulated on thymic T_regs_ [34]. Cells from blood were analysed by flow cytometry over the course of CIA development. Even though the overall proportions of CD4^+^CD25^+^ T cells were similar between groups (data not shown), there was an increase in CD4^+^CD25^+^FoxP3^+^ Helios^+^ T cells at days 35 and 42 in LNT-Ii-CII treated animals compared to LNT-Ii-CLIP controls (Figures 4(d) and 4(e)). This experiment shows that lentiviral treatment in manifest arthritis, causing overexpression of the CII epitope, ameliorates the joint pathology which coincides with an expanded thymus derived CD4^+^CD25^+^FoxP3^+^ Helios^+^  T_reg_ cell compartment and a decreased production of antigen-specific antibodies from B cells.

## 4. Discussion

This report represents the first successful attempt to use systemic lentivirus-based gene therapy to induce antigen-specific tolerance in the inflammatory phase of arthritis in mice. Importantly, a single i.v. injection of LNT-Ii-CII lentiviral particles—particularly early after CII immunisation (day 7) and around onset of arthritis (day 26), but also to some extent in manifest disease (day 31)—reduced the severity of arthritis, measured both by clinical and histopathological evaluation. This partial prevention of arthritis was accompanied by an increased suppressive capacity by T_regs_ on T effector cells as well as decreased levels of CII-specific IgG antibodies.

Treatment by gene therapy in the effector phase of arthritis is a continuation of our previous study in which we showed that prophylactic therapy, administered i.v. 28 days prior to CIA immunisation, decreased severity and frequency of arthritis [30]. Since most protocols for induction of tolerance in CIA have been performed using repeated administrations of antigen to achieve a treatment effect [26, 35], it is striking that a single injection of tolerogenic lentiviral particles rendered a substantial amelioration of arthritis particularly when given at days 7 or 26 after CII immunisation. This is in line with the study by Dzhambazov et al. which showed that vaccination with the CII epitope complexed with soluble A^q^ molecules has both prophylactic and therapeutic effects in a model of chronic CIA [26]. Treatment of mice with manifest arthritis (day 31) was less efficient although histopathological analysis clearly showed reduced bone-cartilage erosivity. This is likely to be the strongest result achievable in the DBA/1 model as these mice do not develop chronic inflammatory arthritis and we can only affect the remaining active inflammation at this late stage.

The development of CIA is dependent on both T and B cells [36–38] and in part believed to be controlled by T_regs_ [39–43]. Earlier studies have shown that the proportion T_regs_ (CD4^+^CD25^+^Foxp3^+^) is increased, or at least not decreased, during various autoimmune conditions both in mouse models and their human counterparts [42–46]. It has also been suggested that their function is impaired either due to genetic predisposition or due to the inflammatory environment in which they act [47, 48]. Studies on transfer of T_regs_ as treatment in CIA have shown a good effect during the early stage of arthritis while being more inefficient during later stages of CIA [49]. TNF-blocking therapies have shown a restoring effect on impaired T_regs_ in RA [50]. The transcription factor Helios can be used as a marker for thymic-derived T_regs_ [34]; however, later studies have shown that Helios expression also can be an activation marker for T effector cells, particularly in humans [51]. As the T cell subset we analysed was positive for several T_reg_ markers such as CD25^high^ and FoxP3 in addition to Helios, this suggests that the T cells induced by LNT-Ii-CII treatment actually belong to the T_reg_ cell compartment and might be of thymic origin and participate in the control of arthritis. We found that the frequency of CD4^+^CD25^+^Foxp3^+^  T_regs_ was similar in the treatment and control groups in lymph nodes and spleen, while their suppressive capacity on T effector cells was increased after LNT-Ii-CII treatment day 26. Looking closely at T_regs_ in the circulation after LNT-Ii-CII injection at day 31, we found that an increased proportion of these cells coincided with reduced severity of arthritis and also preceded a decrease in CII-specific IgG antibody levels. This implies that differences in the frequency of T_reg_ subsets can play an important role in the control of arthritis development both directly and indirectly via reduced production of CII-specific IgG antibodies.

It is well known that CII-specific antibodies are pivotal for the development of CIA [38] and also seem to predict a more erosive form of human RA [52]. CII-specific IgG antibodies have multiple effects in arthritis; they can have a cartilage destabilizing function and subsequently contribute to inflammation and bone-cartilage erosivity through immune complex formation and/or activation of complement [53–56]. The effect seen on CII-specific antibody production in our experiments is not due to a general reduction of IgG levels, since IgG levels in serum were comparable or even increased in LNT-Ii-CII treated animals compared with controls. However, we cannot rule out that the effects observed on T_regs_ and B cells is not due to an effect of amelioration of arthritis *per se* and that treatment with LNT-Ii-CII suppresses arthritis via other mechanisms.

## 5. Conclusion

LNT-Ii-CII treatment in various phases of CIA ameliorates arthritis and is accompanied by a profound effect on both the T cell and B cell compartments in an inflammatory environment. A shift towards tolerance might thus be possible even during the inflammatory phase of arthritis. However, to be able to establish tolerance in human RA the immunological mechanisms involved need to be clarified in further detail.
